# Early blood neurofilament light chain and myelin oligodendrocyte glycoprotein antibody levels associate with different disease courses of myelin oligodendrocyte glycoprotein-associated disease in children

**DOI:** 10.1093/braincomms/fcad063

**Published:** 2023-03-15

**Authors:** Philippe Horellou, Lorraine Flet-Berliac, Carole Leroy, Laetitia Giorgi, Candie Joly, Delphine Desjardins, Pascale Chrétien, Salima Hacein-Bey-Abina, Roger Le Grand, Kumaran Deiva

**Affiliations:** Center for Immunology of Viral, Auto-Immune, Hematological and Bacterial diseases (IMVA-HB/IDMIT), Université Paris-Saclay, CEA, INSERM, Le Kremlin Bicêtre 94276, France; Center for Immunology of Viral, Auto-Immune, Hematological and Bacterial diseases (IMVA-HB/IDMIT), Université Paris-Saclay, CEA, INSERM, Le Kremlin Bicêtre 94276, France; Center for Immunology of Viral, Auto-Immune, Hematological and Bacterial diseases (IMVA-HB/IDMIT), Université Paris-Saclay, CEA, INSERM, Le Kremlin Bicêtre 94276, France; Center for Immunology of Viral, Auto-Immune, Hematological and Bacterial diseases (IMVA-HB/IDMIT), Université Paris-Saclay, CEA, INSERM, Le Kremlin Bicêtre 94276, France; Center for Immunology of Viral, Auto-Immune, Hematological and Bacterial diseases (IMVA-HB/IDMIT), Université Paris-Saclay, CEA, INSERM, Le Kremlin Bicêtre 94276, France; Center for Immunology of Viral, Auto-Immune, Hematological and Bacterial diseases (IMVA-HB/IDMIT), Université Paris-Saclay, CEA, INSERM, Le Kremlin Bicêtre 94276, France; Clinical Immunology Laboratory, Groupe Hospitalier Universitaire Paris-Saclay, Hôpital Kremlin-Bicêtre, Assistance Publique-Hôpitaux de Paris, Le-Kremlin-Bicêtre 94275, France; Unité des technologies Chimiques et Biologiques pour la Santé, Université de Paris, CNRS, INSERM, UTCBS, Paris F-75006, France; Clinical Immunology Laboratory, Groupe Hospitalier Universitaire Paris-Saclay, Hôpital Kremlin-Bicêtre, Assistance Publique-Hôpitaux de Paris, Le-Kremlin-Bicêtre 94275, France; Unité des technologies Chimiques et Biologiques pour la Santé, Université de Paris, CNRS, INSERM, UTCBS, Paris F-75006, France; Center for Immunology of Viral, Auto-Immune, Hematological and Bacterial diseases (IMVA-HB/IDMIT), Université Paris-Saclay, CEA, INSERM, Le Kremlin Bicêtre 94276, France; Center for Immunology of Viral, Auto-Immune, Hematological and Bacterial diseases (IMVA-HB/IDMIT), Université Paris-Saclay, CEA, INSERM, Le Kremlin Bicêtre 94276, France; Pediatric Neurology Department, Assistance Publique-Hôpitaux de Paris, Paris-Saclay University Hospitals, Bicêtre Hospital, Le Kremlin Bicêtre 94270, France; National Referral Center for Rare Inflammatory and Auto-Immune Brain and Spinal Diseases (MIRCEM), AP-HP Le Kremlin Bicêtre 94270, France

**Keywords:** neuroinflammation, autoimmune diseases, acquired demyelinating syndromes, myelin oligodendrocyte glycoprotein, neurofilament

## Abstract

Acquired demyelinating syndrome associated with myelin oligodendrocyte glycoprotein antibodies, named recently myelin oligodendrocyte glycoprotein-associated disease, represents >27% of this paediatric syndrome. Relapses occur in 40% of them, which may be associated with severe outcomes. Aiming to identify biomarker allowing to predict relapse, we measured both myelin oligodendrocyte glycoprotein antibodies and neurofilament light chain levels in blood samples of patients that are known to reflect axonal injuries in neurological diseases including demyelinating autoimmune disorders. Three groups of patients were selected: relapsing myelin oligodendrocyte glycoprotein-associated disease (*n* = 8), non-relapsing myelin oligodendrocyte glycoprotein-associated disease (*n* = 7) and control patients with non-inflammatory neurological diseases (*n* = 12). Neurofilament light chain concentrations were measured in plasma of these three groups of patients using the high-sensitivity single-molecule array method at onset of the disease and 6 months later. At onset of the disease, we found that levels of neurofilament light chain in blood of non-relapsing patients were significantly higher than in control patients (means: 98.36 ± 22.66 versus 12.47 ± 2.47 pg/mL, **P < 0.01, Kruskal–Wallis test). The mean neurofilament light chain value in relapsing patients (82.16 ± 38.41 pg/mL) was not significantly different from that in non-relapsing and in control patients. Plasma myelin oligodendrocyte glycoprotein antibody levels were 2.5-fold higher in relapsing than in non-relapsing patients without reaching significance (means: 15.26 ± 4.87 versus 5.96 ± 1.13; two-tailed Mann–Whitney U-test *P* = 0.119). Plasma neurofilament light chain correlated significantly with myelin oligodendrocyte glycoprotein antibody levels in relapsing (two-tailed Spearman *r* = 0.8, *P* = 0.0218) but not in non-relapsing (two-tailed Spearman *r* = 0.17, *P* = 0.71). Interestingly, the ratio of neurofilament light chain-to-myelin oligodendrocyte glycoprotein antibodies was significantly lower in relapsing than in non-relapsing patients (means: 5.19 ± 1.61 versus 21.87 ± 6.13; two-tailed Mann–Whitney U-test *P* = 0.014). These findings suggest that measuring both neurofilament light chain and myelin oligodendrocyte glycoprotein antibody levels in patients at onset of demyelinating disease could predict relapse of myelin oligodendrocyte glycoprotein-associated disease.

## Teaser

Horellou *et al*. found that blood neurofilament light chain levels at onset in myelin oligodendrocytes glycoprotein (MOG)-associated disease in children correlate with anti-MOG antibody levels and suggest that comparing neurofilament and MOG antibody levels could allow to predict relapse of MOG-associated disease, potentially associated with severe outcomes.

## Graphical Abstract

**Graphical Abstract:**
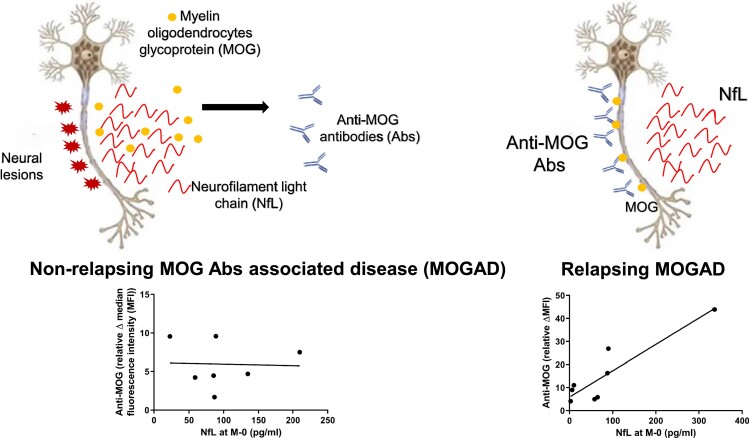

## Introduction

Paediatric acquired demyelinating syndromes (ADS) are rare immune-mediated acute demyelinating disorders of the central nervous system (CNS) with an incidence of 0.6–1.6 for 100 000 children per year in western countries.^1-3^ Myelin oligodendrocyte glycoprotein (MOG) antibodies (Abs) are found in >27% of paediatric ADS that are referred to as MOG antibody-associated disease (MOGAD).^4^ Relapses occur in 40% of them, which may be associated with severe outcomes.^5^ MOG protein, which represents only 0.05% of myelin proteins, is expressed exclusively on the outer surface of the myelin sheath and the plasma membrane of oligodendrocytes. Its cell surface location makes it accessible to immune reactions^6^ and is a target of autoimmune responses that cause inflammation and CNS demyelination.^7,8^ In MOG-induced experimental allergic encephalomyelitis (EAE) in mice, an irreversible axonal loss is detected in lesions at an early stage.^9,10^ In this model, in spinal cord, the density of leukocytes, T cells and damaged axons increased from the onset to the peak phase.^11^ It was suggested that in the early acute stage in MOG-EAE in mice, neurodegeneration might be triggered by inflammatory activity.^9^

Recently, by studying response of blood lymphocytes to recombinant human MOG (rh-MOG), we found immunological differences between relapsing and non-relapsing MOGAD (MOGNR) children at onset of demyelinating events. We observed a significant increase of CD4^+^ lymphocyte T helper 17 (Th17) induced by rh-MOG stimulation in patients without relapse (MOGNR) at onset of the disease. CD4^+^Foxp3^+^ T_regs_ were significantly increased in response to rh-MOG in MOGNR, while CD45RA^−^Foxp3^+^ T_regs_ decreased upon rh-MOG stimulation in relapsing MOGAD (MOGR) patients.^12^ It was suggested that MOG Abs and MOG-autoreactive lymphocytes might be involved in MOG Abs-associated demyelination^13^ and axonal damage.^14^ We therefore decided to evaluate axonal injuries using biological markers in MOGR and MOGNR. In this aim, we made use of neurofilament light chain (NfL) levels in plasma and serum of patients, a relevant biomarker for axonal injury in neurological diseases, quantified by an ultrasensitive single-molecule array (Simoa) assay recently developed.^15^

NfL, a specific element of the neuron cytoskeleton localized abundantly in axons, is released in the extracellular space after neuronal injury.^16^ In children with no neurologic disorder, high levels of blood NfL are found in newborn infants, followed by a decrease until the age of 10 and 15 years, marked by a nadir.^17-19^ Afterward, blood NfL levels are rising in a linear manner until 60 years of age, followed by a more steeper increase,^17,18^ probably reflecting age-related neuronal degeneration.^18,20,21^ There is no gender effect on serum NfL,^18,20^ even though cerebrospinal fluid (CSF) NfL is higher in men by 26%.^21^ A close association of increased blood NfL levels with neuronal damage has been suggested in demyelinating disease such as multiple sclerosis, with focal lesions presence in both brain and spinal cord, as well as in other neurological conditions, including amyotrophic lateral sclerosis, neurodegenerative disorders and acute brain and spinal cord injury.^20,22^ In relapsing–remitting multiple sclerosis, NfL appears as promising biomarkers of disease activity and treatment response, while in progressive multiple sclerosis, their implication is yet unclear.^23^ A recent observational study found that, in multiple sclerosis, NfL levels were closely associated with clinical and radiological activity and that most of individuals with high NfL levels as the only evidence of disease activity had progressive multiple sclerosis.^24^ Other authors found greater levels of NfL in relapsing–remitting multiple sclerosis than in progressive multiple sclerosis.^25^ A systematic review suggested that differences in NfL between relapsing–remitting multiple sclerosis and progressive multiple sclerosis could be explained by differences in covariates.^23^ NfL may also increase in relation to peripheral neuropathy.^26^ Following axonal damage, NfL levels are released in large quantities in the CSF, and eventually into the blood, where concentrations are 42-fold lower than in the CSF.^20,27^

Assay of this biomarker in the CSF, but also in blood, allows to monitor the impact of ADS on neurons and to follow effects of treatment.^28^ In children with ADS, a significant correlation between CSF and serum NfL was found that strengthens its reliability as a peripheral marker of neuroaxonal damage.^29^ Serum NfL was higher in paediatric ADS patients than in controls and was highest in ADS with acute demyelinating encephalomyelitis (ADEM).^29^ In MOGAD in adult patients, it was shown that serum NfL levels increase, suggesting the concomitant presence of myelin and axonal damage in this disorder.^30^ In these patients, serum NfL levels correlate with attack severity and might predict long-term outcome.^31^ More recently, NfL levels in adult MOGAD patients were found to be higher at onset, whereas relapses were not associated with an axonal burden as compared to that detected at disease onset.^32^ This observation confirmed the concept that in MOGAD, disability mainly derives from the onset attack, as previously shown in clinical studies.^33,34^

We herein measure blood NfL levels at onset and at 6-month follow-up of ADS patients and evaluate differences between relapsing and MOGNR children beside control patients in relation to MOG Abs levels.

## Methods

### Patients and controls

Fifteen children aged ≤18 years from the French cohort Kidbiosep composed of patients followed after a first demyelinating event in the National Referral Center for Rare Inflammatory and Auto-Immune Brain and Spinal Diseases at Bicêtre Hospital, from January 2011 to May 2018, were involved in this study. ADS was characterized by an acute neurological deficit lasting longer than 24 h affecting the CNS, the optic nerve, brain, cerebellum, brainstem and/or spinal cord, in relation to *T*_2_ lesions on magnetic resonance imaging. Recurrence was defined as a new CNS demyelination event at least 1 month after the first one or 3 months if the first attack is an ADEM and lasts at least 24 h without fever or infection. All patients received steroid therapy by oral prednisone of 1 mg/kg/day during the first month and then taper over the second month. Demographic data of patients are presented in Table 1. The diagnosis of MOGAD was made according to the international consensus diagnostic criteria for neuromyelitis optica spectrum disorders.^35^ We gathered patients included within three groups: MOGNR patients (*n* = 7), MOGR patients (*n* = 8) and control children (CTRL, *n* = 12). Control patients involved were from Paediatric Neurology Department at Bicêtre Hospital hospitalized for non-inflammatory neurological diseases, such as benign intracranial hypertension (*n* = 3), cognitive deficit (*n* = 3), post-infectious encephalitis (*n* = 1), dysaesthesia (*n* = 1), moyamoya disease (*n* = 1), walking ability transient disorder (*n* = 1), autism (*n* = 1) and spondylolisthesis (*n* = 1) and had blood samples collected for diagnosis.

**Table 1:** Demographic data of included children

	MOGR *n* = 8	MOGNR *n* = 7	Controls *n* = 12	*P*-value
Female (*n*, %)	6 (75)	3 (43)	3 (25)	0.3
Age at onset (mean, years ± SD)	5.8 ± 3.5	6.8 ± 3.8	9.8 ± 4.1	0.08
Age at onset for MOGAD (*n* = 15)	6.3 ± 3.5			0.051
Timing of relapses (mean, years ± SD)	1.9 ± 2.1			
Presentation				
Optic Neuritis	1	1		0.99
ADEM	2	3		0.58
Non-ADEM encephalitis	2	2		0.99
NMOSD	3	1		0.58
EDSS at onset (mean ± SD)	0.12 ± 0.35	1 ± 2.64		0.73
CSF pleocytosis (≥20 cells/mm^3^ (*n*, %)	3 (37)	3 (43)		0.99
CSF proteins (≥0.5 g/dL) (*n*, %)	1 (12)	1 (14)		0.99
Follow-up time (mean, years ± SD)	8.6 ± 4.8	4.5 ± 2.7		0.095

Data are expressed as mean ± standard deviation (SD) unless otherwise stated. For age at onset and follow-up time, one-way ANOVA and Mann–Whitney U-test *P*-values are given, respectively. For age at onset for MOGAD versus controls, Mann–Whitney U-test *P*-value is given. For gender, the chi-square *P*-value is given. For EDSS, Mann–Whitney U-test *P*-value is given. For all other values, the Fisher’s exact test *P*-values are given. Controls, other non-inflammatory neurological disorder patients; MOGNR, non-relapsing MOGAD; MOGR, relapsing MOGAD.

### Ethics

This study complied in all respects with French national and local ethics committee guidelines. The French national cohort of first demyelinating episode ‘Kidbiosep 2004’ (No. 910506) received authorization from the Commission Nationale de l'Informatique et des Libertés and the Comité de Protection des Personnes of Paris-Saclay University. An informed consent form was signed by parents of each child involved in the study.

### Blood samples

Blood samples were collected on lithium heparin tubes from all patients at their first demyelinating episode, within the first 3 months and before starting immunosuppressive or immunoregulatory therapy, and again 6 months later for all MOGAD patients, except one MOGNR patient for whom blood sample was not available at 6 months.

### Cell-based assay for detection of Abs to cell surface MOG in plasma

HEK293A cells transfected with a full-length human MOG cDNA expression plasmid were used as antigenic substrate in combination with control non-transfected cells as previously described.^36^ This method was chosen as it is reliable and allows quantification. Briefly, stable MOG-transfected cells were used to assay MOG immunoglobulins in patient plasma by flow cytometry. Non-transfected HEK293 cells were used as control for each sample. Cells grown on plates were detached using phosphate-buffered saline (PBS) with 0.2 mM EDTA, rinsed in 2% foetal bovine serum (FBS)/PBS and blocked with 10% FBS/PBS during 1 h at 4°C. About 150 000 cells were incubated with patient plasma at 1:2 and 1:5 dilutions for 1 h at 4°C, followed by three washes with 2% FBS/PBS, and incubation with fluorescein isothiocyanate (FITC) conjugated anti-human immunoglobulin anti-IgG H + L Fab'2 secondary antibody (Kallestad FITC conjugate, Bio-Rad, Marnes la Coquettes, France) for 15 min at 4°C. After a last wash, cells were fixed and resuspended in 300 μL of 2% formaldehyde PBS before analysis. For each sample, 50 000 events were recorded and analysed on a FACS Canto II (BD Biosciences). Flow cytometric analysis was performed using FlowJo software (Ashland, OR, USA) and Excel. Levels of specific antibody binding on MOG-transfected cells were expressed as Δ median fluorescence intensity (MFI), determined by subtracting MFI measured on HEK293 control cells from the MFI measured on HEK293MOG^+^ cells. A ΔMFI greater than mean + 6 standard deviations (SDs) of values of the control patients’ samples was considered positive. Each assay was performed twice. Positive plasmas were then assayed by serial dilution from 1:10 to 1:640 with a threshold of 1:160 to define positivity. For quantification, we used the MOG ratio of ΔMFI, calculated with the formula (MFI MOG-HEK293 cells − MFI HEK293 cells)/MFI HEK293 cells, considered as an alternative and straightforward method to determine MOG Abs levels.^37^

### Measurements of NfL concentrations with Simoa technology

Plasma samples were collected from each patient and frozen at −20°C. NfL concentrations were measured with Simoa NF-Light Advantage Kit (Quanterix, Lexington, MA) according to the manufacturer’s instructions. The assay was carried out on Simoa HD-X Analyser (Quanterix Corporation, Lexington, MA, USA). We analysed all samples in duplicate within one assay. The mean intra-assay coefficient of variation of duplicate determinations for concentration ranged from 3% to 6%.

### Statistical analysis

We performed statistical analyses using Graph Prism 8® software. Data are presented as mean ± standard error of the mean (SEM) as indicated. Statistical analyses were performed by Mann–Whitney U-test or Kruskal–Wallis test followed by Dunn post-test correction. Statistical significance was assigned to values of *P* < 0.05, and the symbols used were *P* < 0.05 (*), *P* < 0.01 (**) and *P* < 0.001 (***). Wilcoxon signed rank test at 95% confidence interval (CI) was used for statistical evaluation of patients at onset and at 6 months later.

## Results

### Demographic characteristics of patients

Demographic data are summarized in Table 1. Twenty-seven children were included in this study. The mean onset age of each group was similar as analysed by one-way ANOVA. The gender proportion of each group was similar as analysed by chi-square test. The mean Expanded Disability Status Scale (EDSS) score measured at onset was 0.12 ± 0.35 in MOGR and 1 ± 2.64 in MOGNR without reaching significance (two-tailed Mann–Whitney U-test *P* = 0.73).

### NfL concentrations in plasma

We measured NfL levels in plasma of patients, since it is increased in relation to axonal damage in particular in demyelinating disorders in association with neuroinflammation^38^ and as it is considered as a quantitative measure of neuronal loss rate in the CNS at the time of sampling.^39^

**Figure 1:**
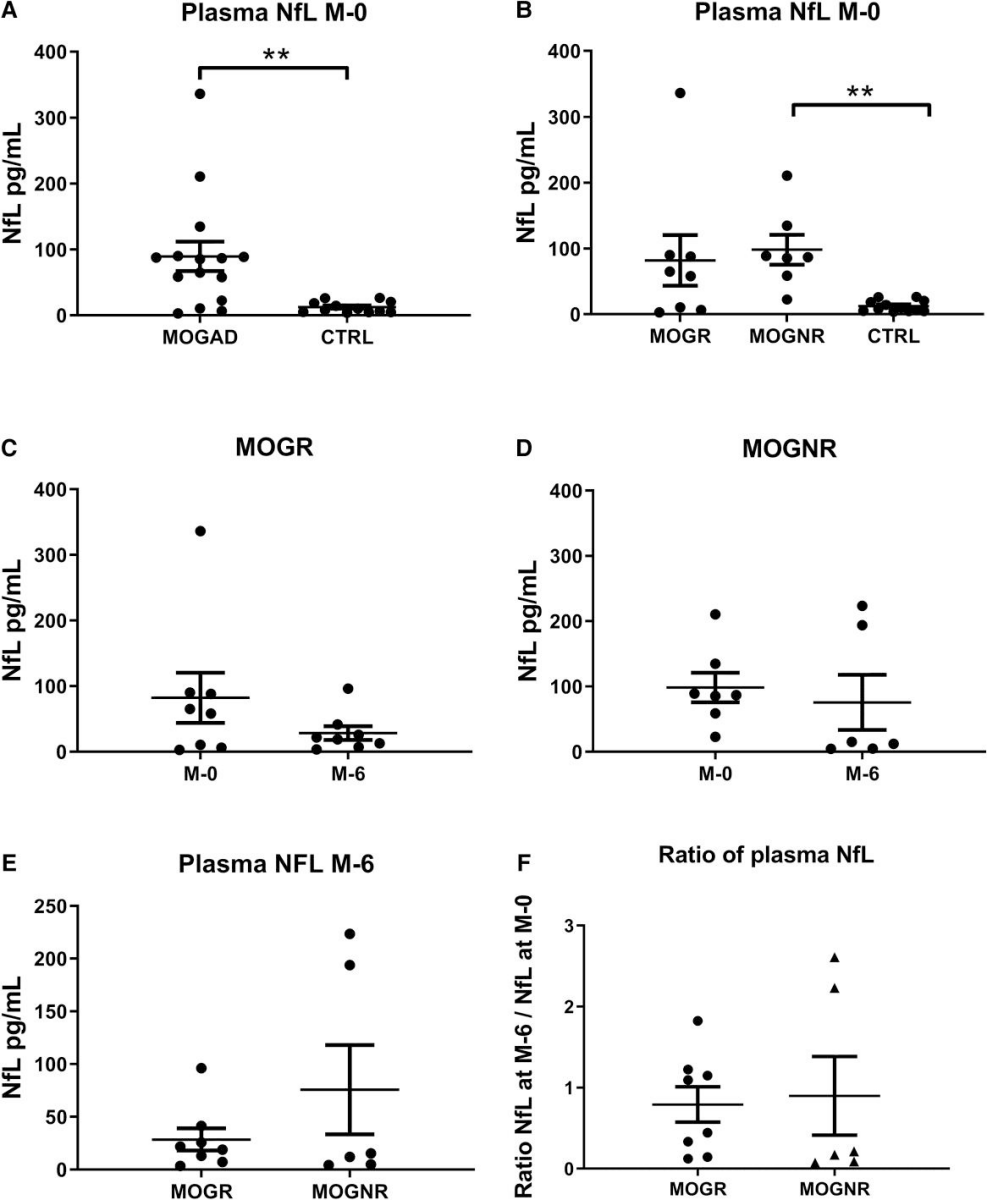
**Blood NfL is higher in MOGNR at onset.** The levels of NfL (pg/mL) are shown in MOGAD and in CTRL (**A**) as well as in relapsing ADS with MOG Abs (MOGR) and non-relapsing ones (MOGNR) and controls patients (CTRL) (**B**), and (**E**) 6 months later in MOGR and MOGNR. For comparison, we show on the same graph levels of NfL at onset and 6 months later in MOGR (**C**) and in MOGNR (**D**). (**F**) Ratio of blood NfL at 6 months to blood NfL at onset in MOGR and MOGNR groups of patients. Means and SEM values are indicated. Two-tailed Mann–Whitney U-test (**A**) and Kruskal–Wallis and Dunn correction (**B**) **P < 0.01.

Mean NfL values were significantly higher in MOGAD than in control patients (means: 89.72 ± 22.38 versus 12.47 ± 2.47 pg/mL, two-tailed Mann–Whitney U-test *P* = 0.0012; Fig. 1A). When separating MOGAD into MOGNR and MOGR subgroups, we found that mean NfL value measured at onset in MOGNR patients was significantly higher than that in control patients (means: 98.36 ± 22.66 versus 12.47 ± 2.47 pg/mL, **P < 0.01, Kruskal–Wallis and Dunn correction). The mean NfL value in MOGR (82.16 ± 38.41 pg/mL; Fig. 1B) was not significantly different from that in MOGNR and in control patients.

At 6-month post-onset, NfL levels were higher in MOGNR than in MOGR without reaching significance (means: 75.65 ± 42.27 versus 28.46 ± 10.53 pg/mL, two-tailed Mann–Whitney U-test *P* = 0.903; Fig. 1E). This is related to two patients in the MOGNR group that remained high and had monophasic ADEM. From onset values, the mean NfL values decreased by 6 months without reaching significance in MOGR group (two-tailed Wilcoxon test *P* = 0.547; Fig. 1C) and in MOGNR (two-tailed Wilcoxon test *P* > 0.9; Fig. 1D) groups. When analysing the ratio of NfL at 6 months to NfL at onset, there were no significant differences between MOGNR and MOGR groups (means: 0.90 ± 0.48 versus 0.79 ± 0.22; two-tailed Mann–Whitney U-test *P* = 0.632; Fig. 1F), suggesting that the decrease was similar in MOGNR and in MOGR groups.

### MOG Abs levels and relations with NfL levels

**Figure 2:**
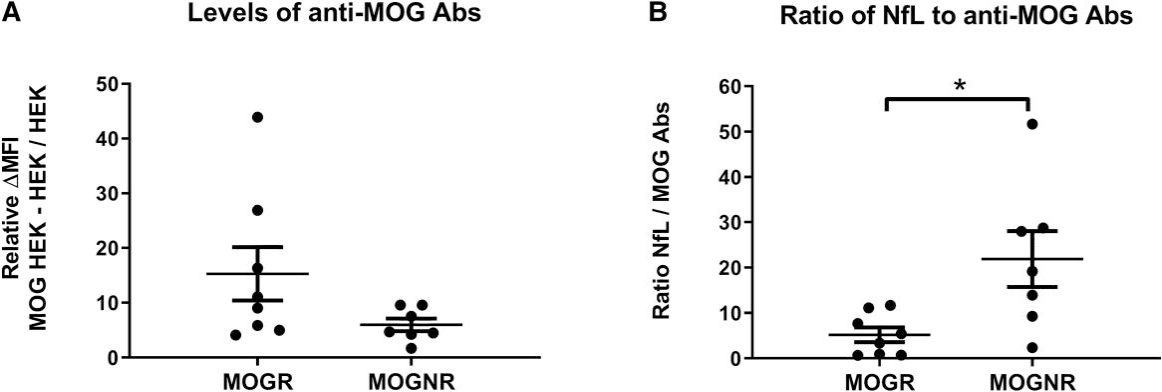
**Levels of anti-MOG antibodies at onset in MOGR and MOGNR.** Onset levels of anti-MOG antibodies, expressed as relative ΔMFI and calculated as (MFI MOG-HEK293 cells − MFI HEK293 cells)/MFI HEK293 cells, are shown in MOGR and in MOGNR (**A**). Ratio of NfL levels to anti-MOG Abs levels at onset is shown in MOGR and MOGNR (**B**). Means and SEM values are indicated. Two-tailed Mann–Whitney U-test *P < 0.05.

Levels of anti-MOG Abs were 2.5-fold higher in MOGR than in MOGNR group of patients at onset of the disease without reaching significance (means: 15.26 ± 4.87 versus 5.96 ± 1.13; two-tailed Mann–Whitney U-test *P* = 0.119; Fig. 2A).

### NfL-to-MOG Abs ratio is higher in MOGNR than in MOGR

Since NfL is higher in MOGNR than in MOGR, contrary to MOG Abs levels, we decided to evaluate the relative importance of these two markers in the two groups of patients. We therefore analysed the ratio of NfL to MOG Abs levels and found that it was significantly lower in MOGR than in MOGNR (means: 5.19 ± 1.61 versus 21.87 ± 6.13; two-tailed Mann–Whitney U-test *P* = 0.014; Fig. 2B).

### MOG Abs correlate with NfL levels in MOGR

**Figure 3:**
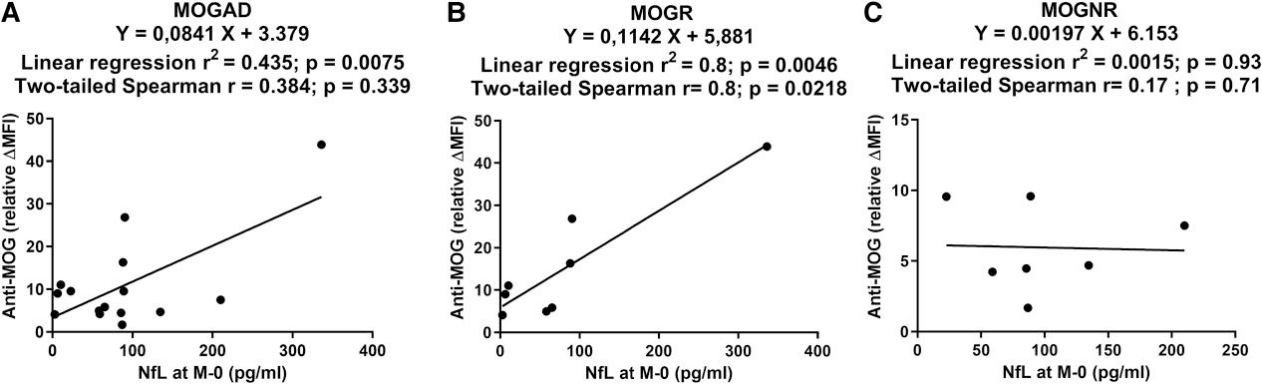
**Anti-MOG antibodies correlate with NfL in MOGR.** Linear regressions between relative ΔMFI and NfL at onset of the disease are presented in MOGAD (**A**), MOGR (**B**) and MOGNR (**C**). Linear regression correlation coefficients and corresponding *P*-values (two-tailed Pearson) as well as rank correlation coefficients and corresponding *P*-values (two-tailed Spearman) are given.

We then evaluated whether there was a relation between MOG Abs and NfL levels at onset in MOGAD and in relapsing and non-relapsing forms of the disease by conducting linear regression between NfL and MOG Abs levels. In MOGAD, there was a significant linear correlation (corresponding to Pearson’s analysis) without significant rank correlation (corresponding to Spearman’s analysis) between MOG Abs and NfL levels at onset (linear regression *R*^2^ = 0.435; *P* = 0.0075; two-tailed Spearman *r* = 0.384, *P* = 0.339; Fig. 3A). In MOGR subgroup, there was a significant linear correlation and a significant rank correlation (linear regression *R*^2^ = 0.8; *P* = 0.0046; two-tailed Spearman *r* = 0.8, *P* = 0.0218; Fig. 3B). On the contrary, there was neither significant linear correlation nor significant rank correlation in MOGNR subgroup (linear regression *R*^2^ = 0.0015; *P* = 0.93; two-tailed Spearman *r* = 0.17, *P* = 0.71; Fig. 3C).

### NfL-to-MOG Abs ratio at onset is associated with the course of MOGAD

**Figure 4:**
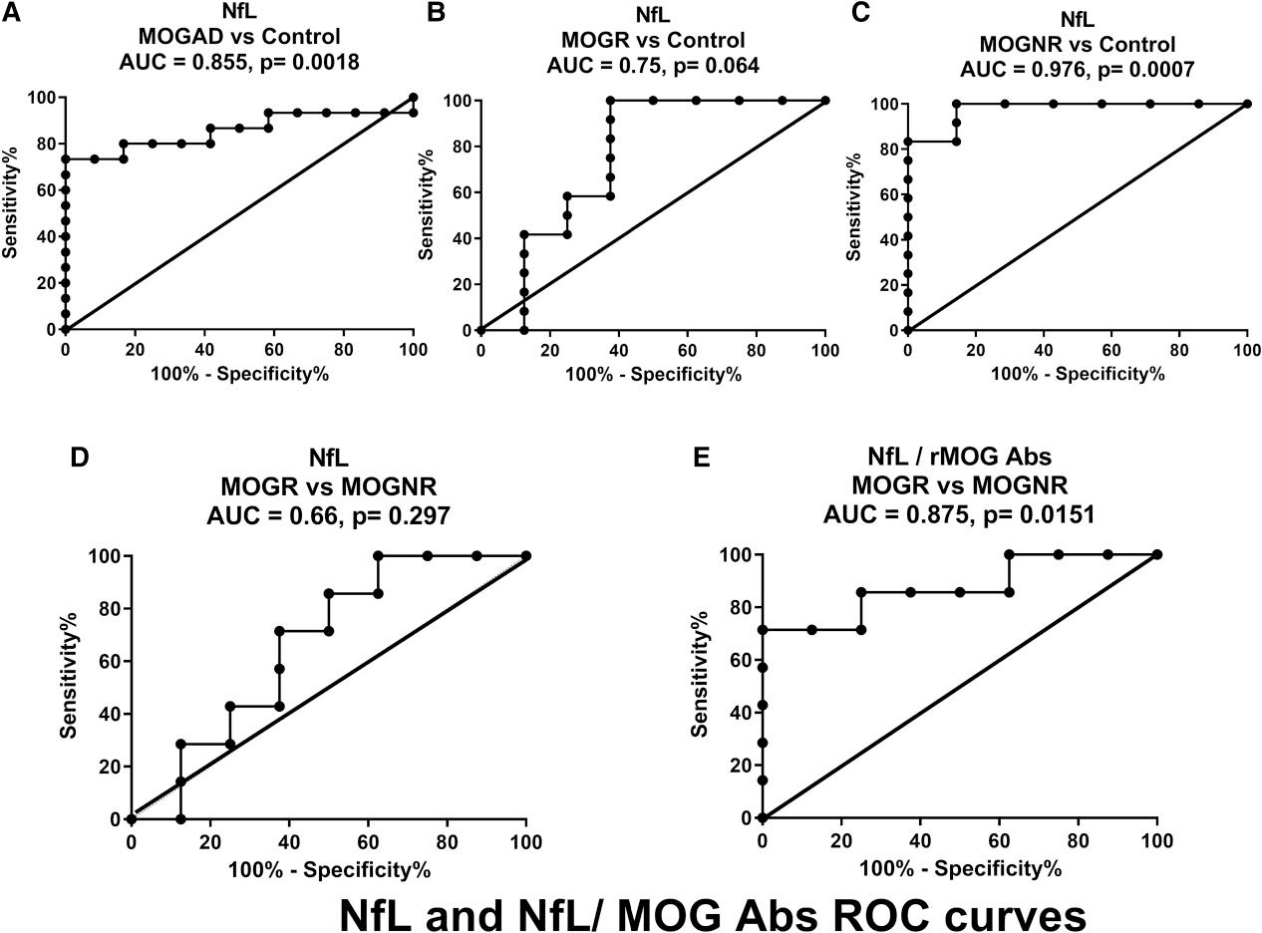
**ROC plots of NfL levels for MOGR.** Empirical ROC curves using NfL levels indicating AUC and *P*-values allowing to differentiate between children diagnosed with MOGAD and control (**A**) and between children diagnosed with MOGNR and control (**C**). ROC curves using NfL levels cannot be used to differentiate between children diagnosed with MOGR and control (**B**) and those with MOGR and MOGNR (**D**) as indicated by *P*-values, unlike ROC curve using NfL/MOG Abs levels that allow to differentiate between children diagnosed with MOGR and MOGNR (**E**).

To evaluate whether NfL could be used as a predicting biomarker of relapse, we performed receiver operating characteristic (ROC) curve analysis of NfL levels at onset among MOGAD and control patients as well as among MOGR, MOGNR subgroups and control patients. MOGAD could be discriminated from control based on NfL levels at onset [area under the ROC curve (AUC) = 0.855; 95% CI 0.7–1.01; *P* = 0.0018; Fig. 4A]. Comparing MOGR subgroup and control group showed that NfL could not be used to discriminate between these groups of patients (AUC = 0.75; 95% CI 0.49–1.01; *P* = 0.064; Fig. 4B). On the contrary, NfL levels at onset allow to discriminate MOGNR subgroup from control patients (AUC = 0.976; 95% CI 0.91–1.03; *P* = 0.0007; Fig. 4C). This test does not allow to discriminate between MOGR and MOGNR patients (AUC = 0.66; 95% CI 0.37–0.94; *P* = 0.297; Fig. 4D). However, when analysing NfL-to-MOG Abs ratio, it was possible to discriminate between MOGR and MOGNR patients (AUC = 0.875; 95% CI 0.68–1.06; *P* = 0.0151; Fig. 4E). By using the Youden index and the point at the shortest distance to the upper left corner of the graph on the MOGAD versus control ROC curve (Fig. 4A), we could define an optimal cut-off value of blood NfL at onset of 42.45 pg/mL with an accuracy of 85% with 0.73 sensitivity and 1 specificity. This method applied to the MOGNR versus control ROC curve (Fig. 4C) allows to define an optimal cut-off value of blood NfL at onset of 42.85 pg/mL with an accuracy of 95% with 1 sensitivity and 0.86 specificity. More interestingly, the same approach for MOGNR versus MOGR ROC curve (Fig. 4E) indicated an optimal cut-off value of blood NfL/MOG Abs at onset of 12.8 relative units with an accuracy of 86% with 0.71 sensitivity and 1 specificity.

## Discussion

In this study, we found that in children at onset of MOGAD, NfL levels in blood were significantly higher than in control patients. Similar results were found in MOGAD adult patients in the study of Mariotto *et al.*,^31^ as well as more recently in paediatric patients.^40,41^ In our MOGNR subgroup of patients, NfL levels were higher than in the MOGR subgroup and significantly higher than in control patients. The higher levels of NfL in MOGNR could be related to the higher neuroinflammation in these patients. Such a hypothesis is corroborated by our recent study of immunological response to rh-MOG as an antigenic stimulation in peripheral blood mononuclear cells (PBMC) from patients. In patients with MOGNR at onset of the disease, we recently found a higher Th17 response to rh-MOG than in patients with MOGR.^12^ Th17 has been associated with demyelinating diseases in children and adults during inflammatory events,^42,43^ in particular in children with MOGAD.^13^ In addition, Th17-related cytokines G-CSF and IL-6 levels are increased in MOGAD,^13^ and IL-6 levels in CSF correlate with MOG antibody levels in paediatric patients with monophasic acquired demyelination syndromes.^36^ At 6 months, the levels of NfL in MOGNR are higher than that in MOGR. We found that this is related to two patients in the MOGNR group that remained high and had monophasic ADEM.

Consistently with the study by Mariotto *et al.*,^31^ we found no direct relation between NfL levels at onset and risk of relapse. However, since in our MOGNR subgroup of patients, NfL levels were higher than in the MOGR subgroup and significantly higher than in control patients, we further analysed the NfL level difference between these groups of patients in relation to time and with MOG Abs levels. In both MOGR and MOGNR subgroups, NfL levels were decreased at 6 months, without reaching significance. Thanks to the use of MOG ratio of ΔMFI, a method to quantify MOG Abs,^37^ we found that their levels were higher in MOGR than in MOGNR, without reaching significance. Other studies have shown that persistent high MOG Abs correlates with relapsing forms.^33,37,44-48^ Interestingly, in our MOGAD patients at onset of their disease, NfL levels correlated significantly with MOG Abs levels. Such a correlation also exists for MOGR patients but not for MOGNR ones.

Because NfL levels are higher in MOGNR than in MOGR, whereas on the contrary, MOG Abs levels are lower in MOGNR than in MOGR, we decided to analyse the ratio of NfL levels to that of MOG Abs in these groups of patients. Interestingly, NfL-to-MOG Abs ratio was significantly higher in MOGNR than in MOGR. It is therefore possible that the ratio of NfL-to-MOG Abs could have a prognostic significance indicating that patient with high NfL-to-MOG Abs ratio may have a monophasic demyelinating disease or MOGNR. Performing such an analysis in a wider cohort of patients will be important to confirm this result. This significant difference might be explained by different mechanisms involved in MOGNR and in MOGR. To explain MOG autoimmunity, it was suggested that a direct infection of the brain could induce leakage of CNS antigens in the periphery, inducing a secondary peripheral immune reaction against MOG^49,50^ or alternatively that a peripheral infection would stimulate MOG antibody production via molecular mimicry.^49,51^ In non-relapsing form of the disease, MOG Abs are likely to be generated secondarily to neural lesions, whereas in relapsing forms, MOG Abs are thought to be directly involved in neural lesions. Hence, in non-relapsing forms, inflammatory lesions of the white matter may induce MOG Abs at low rate as a consequence of the lesions, whereas in relapsing forms, MOG Abs being a cause of the lesions, they are generally expressed at a high rate.

ROC curve analysis among MOGAD, MOGR, MOGNR and control patients reinforces the idea that NfL and MOG Abs can be used as predicting biomarkers of relapse. ROC curves using NfL levels could be used to differentiate between children diagnosed with MOGAD and control patients and between children diagnosed with MOGNR and control patients. Interestingly, ROC curve using ratio of NfL-to-MOG Abs allows to differentiate between children diagnosed with MOGR and MOGNR. Analysis in a wider cohort will allow to evaluate different subsets of MOGNR and MOGR patients, in particular ADEM patients. Such an analysis will also allow to envisage the interest of evaluating NfL values at individual level. In multiple sclerosis, the clinical importance of NfL assay at individual level is illustrated by the fact that response to treatment may be indicated by a reduction of NfL levels.^52-56^ Along this line, the use of NfL in multiple sclerosis is also suggested in clinical trial protocols.^57^ However, both standardization of NfL measurement techniques, including sample collection and assay methods, and well-defined diagnostic and prognostic cut-off levels for healthy individuals and patients are still needed.^58^

One major limitation of our study is the small sample size of MOGAD patients included, but this is inherent to the fact that ADS are rare diseases. The absence of healthy control is also a limitation. Due to these limitations, it will be necessary to confirm this study in a larger population of MOGAD patients.

## Conclusion

In conclusion, measuring both NfL and MOG Abs levels in patients at onset of demyelinating disease could be used to follow the different courses of MOGAD. We found a correlation between blood NfL levels and MOG Abs in MOGAD and in MOGR and that ratio of NfL-to-MOG Abs level is higher in non-relapsing than in relapsing course of the disease in patients with ADS. A study in a wider cohort of patients is however required to confirm this result.

## Data Availability

The data that support the findings of this study are available from the corresponding author upon reasonable request.

## Abbreviations

Abs =: antibodies
ADEM =: acute demyelinating encephalomyelitis
ADS =: acquired demyelinating syndromes
MFI =: median fluorescence intensity
MOG =: myelin oligodendrocyte glycoprotein
MOGAD =: MOG antibody-associated disease
MOGR =: relapsing MOGAD
MOGNR =: non-relapsing MOGAD
NfL =: neurofilament light chain
rh-MOG =: recombinant human MOG
Simoa =: single-molecule array
